# How learning can change the course of evolution

**DOI:** 10.1371/journal.pone.0219502

**Published:** 2019-09-05

**Authors:** Leonel Aguilar, Stefano Bennati, Dirk Helbing

**Affiliations:** Professorship of Computational Social Science, ETH Zürich, Zürich, Switzerland; Pavol Jozef Safarik University in Kosice, SLOVAKIA

## Abstract

The interaction between phenotypic plasticity, e.g. learning, and evolution is an important topic both in Evolutionary Biology and Machine Learning. The evolution of learning is commonly studied in Evolutionary Biology, while the use of an evolutionary process to improve learning is of interest to the field of Machine Learning. This paper takes a different point of view by studying the effect of learning on the evolutionary process, the so-called Baldwin effect. A well-studied result in the literature about the Baldwin effect is that learning affects the speed of convergence of the evolutionary process towards some genetic configuration, which corresponds to the environment-induced plastic response. This paper demonstrates that learning can change the outcome of evolution, i.e., lead to a genetic configuration that does not correspond to the plastic response. Results are obtained both analytically and experimentally by means of an agent-based model of a foraging task, in an environment where the distribution of resources follows seasonal cycles and the foraging success on different resource types is conditioned by trade-offs that can be evolved and learned. This paper attempts to answer a question that has been overlooked: whether learning has an effect on what genotypic traits are evolved, i.e. the selection of a trait that enables a plastic response changes the selection pressure on a different trait, in what could be described as co-evolution between different traits in the same genome.

## 1 Introduction

The so called Baldwin effect [1] is a much debated theory in the literature of evolution [2] about how new features are inherited by an individual with phenotypic plasticity [3–5]. Baldwin proposed this new “factor in evolution” [1] to explain how complex features such as an eye can evolve [6–8], as an alternative to the then-popular Lamarckian evolution which assumed that traits acquired by an individual through phenotypic plasticity would be transferred directly to its offspring’s genome [9]. This idea went unnoticed until the late 1990s, when it caught the interest of the fields of Psychology, in reference to the evolution of human learning, and Computer Science, in reference to evolutionary computation, machine learning, and artificial life. Only from the mid-2000s did the Baldwin effect start taking ground in the field of Evolutionary Biology. [10].

Given the long debate surrounding the Baldwin effect, there are different definitions of it with different levels of generality, e.g. “The Baldwin Effect, states that learned behavior and characteristics at the level of individuals can significantly affect evolution at the level of species” [11], Schull relates the Baldwin effect to statements such as “individual developmental responses will necessarily lead to directed and non-random evolutionary change” [12]. The open peer commentaries of [12] highlight different conflicting stances regarding the Baldwin effect and its definition. The working definition of the Baldwin effect used in this paper is: plasticity is a “positive driving force of evolution” that affects the selection pressure such that “standing genetic variation can be selected upon so that evolution can proceed in the direction of the induced plastic response” [8]. According to this definition, the Baldwin effect describes the evolution of a “target” genotypic trait that corresponds to the environment-induced plastic response at the phenotypic level. In other words, the induced plastic response determines the direction towards which the genotype evolves. This definition is especially relevant when considering biologically inspired optimization techniques [13].

A well-known example of the Baldwin effect is that learning, i.e. an instance of phenotypic plasticity, affects the evolutionary process by either speeding up or slowing down the evolution of the “target” genetic configuration.

This work demonstrates that this definition is too restrictive, as a genotypic trait is shown to evolve that differs from the environment-induced plastic response. The term *Baldwin veering effect* is introduced to refer to this new finding and defined as follows: a change in the selection pressure of genetic variations, caused by phenotypic plasticity and induced plastic responses, leads evolution in a different direction from that indicated by the induced plastic response. In other words, the Baldwin veering effect happens when a trait evolves by effect of plasticity that does not correspond to the environment-induced plastic response.

In order to demonstrate the existence of the Baldwin veering effect, the following two conditions have to be verified:

- A trait evolves that differs from the induced plastic response, i.e. the genome and the phenotype converge towards different trait values.
- The evolution of such trait is caused by plasticity, i.e. the genome converges towards different trait values in presence or absence of plasticity.

The effect of plasticity—we choose learning among many potential mechanisms, e.g. polyphenism [14]—on evolution is studied both computationally by means of an agent-based model of a foraging task, modeled after previous work [15, 16], and analytically by means of a mathematical model [17]. Computational experiments and analytical results in a cyclically-changing environment demonstrate the existence of both the Baldwin effect and of the Baldwin veering effect. Specifically, it is found that in a quickly-changing cyclical environment, learning agents evolve a generalist foraging strategy that allows them to adapt quickly to changes in the resource distribution. A generalist configuration is never induced, i.e. learned, at the phenotypic level. Analytical results confirm that plasticity changes the fitness landscape in a way that makes a generalist configuration a global optimum in the space of genotypes.

The novelty of this result is to expand the understanding of the effect of plasticity on evolution by demonstrating that plasticity can affect both the speed and the outcome of evolution. A fundamental difference of this result from previous work [14, 18, 19] is that learning is not only shown to change the phenotype but the genotype as well.

The main contributions of this paper are to show that in a cyclically-changing environment: (I) the well-known Baldwin effect is present, (II) the novel *Baldwin veering effect* is present, (III) a mathematical model captures this new effect and confirms the experimental findings, and (IV) the existence of this new effect depends only on the relation between the speed of learning and the frequency of change in the environment.

## 2 Methods

The computational model follows the agent-based methodology [20] by studying the interactions of a population of software agents, subject to an evolutionary process [21], that perform a foraging task [15, 22], i.e. search the environment for food in a grid like environment. This model builds on the extensive research in the artificial life community, where software agents have been provided with learning mechanisms [11, 23–26] in an evolutionary context. The time-step driven simulation model is based on previous work [27] and favors simplicity over realism. Modeling realistic entities and ecosystems is outside the scope of this work.

This section provides an overview of the essential components of the computational model, the possible phenomenon occurring at every simulated abstract time-unit, and a detailed description of the environment, agents, decision making, and evaluation metrics. Subsection 2.1 describes the cyclically-changing environment, the resources to be foraged and their seasonality. Subsection 2.2 describes the agent most relevant parameters (aptitude and skill) and the trade-offs that these parameters cause during foraging, the relation between foraging and energy levels, and how energy levels affect fitness, reproduction, and death. Subsection 2.3 details the agent behavior (reactive and learning); additionally, it is explained how reproduction affects the parameters related to the decision making process. Finally, subsection 2.4 defines the measures used to evaluate the agents’ behavior. Table 1 contains an overview of the notation used throughout the description of the model. The results presented in this paper are the outcome of 300 Monte Carlo type simulations for each specific scenario.

**Table 1:** Summary of the mathematical notation used in order of appearance in the text. Notation used for indexes has been slightly abused for the sake of brevity.

Math symbol	Description
$A=\{a_{0},...,a_{N}\}$	The set of all *N* agents ever alive in the simulation
*R* = {*r*_0_, …, *r*_*M*_}	The set of *M* resource types
$F^{t}=\left\{\phi_{i,r}^{t}\in E^{t}:\phi_{i,r}^{t}>0\right\}$	Set of all cells containing resources
Φ	The maximum quantity of resource that any cell can contain
$\phi_{i,r}^{t}\in N_{\geq 0}$	The quantity of resources of type *r* in cell *i* at time *t*
$E^{t}=\left\{\phi_{i,r}^{t}:1\leq i\leq m\times m,r\in R\right\}$	The configuration of the environment at time *t*
$T=\{t\in N_{0,\leq L}\}$	The time steps, *t* of the simulation
$s_{a}^{t}\in \left[0,1\right]\;:\;a\in A^{t}$	The skill level of agent *a* at time *t*
$A^{t}=\{a\in A:a\;\text{is}\;\text{alive}\;\text{at}\;\text{timestep}\;t\}$	The population at time *t*
$f\left(a,t\right):A^{t}\times T\to R$	The fitness function
ϵ∈R	Energy level increased by successful foraging
$g\left(a,s_{a}^{t},r\right):A^{t}\times R_{\geq 0,\leq 1}\times R\to \left\{0,1\right\}$	The foraging success function of agent *a* for resource type *r*
$B\left(a,t\right):A^{t}\times T\to O$	The decision function which determines the behavior of agent *a* at time *t*
*O* = {*o*_1_, …, *o*_*n*_}	The set of *n* possible actions
$P_{f}\left(a,s_{a}^{t},r\right):A^{t}\times T\times R\to \left[0,1\right]$	The probability at time *t* of agent *a* to forage resources of type *r*
$P_{r}\left(a,t,c_{r}\right):A^{t}\times T\to \left[0,1\right]$	The probability of reproduction of agent *a* at time *t*, capped at *c*_*r*_
*c*_*r*_	The normalization constant of reproduction
$P_{d}\left(a,t,c_{d}\right):A^{t}\times T\to \left[0,1\right]$	The probability of death of agent *a* at time *t*, capped at *c*_*d*_
$d\left(a,t\right):A^{t}\times T\to N_{0,\leq L}$	The age function, linearly increasing in time.
*c*_*d*_	The normalization constant of death
$I_{a}^{t}=\{i\in E^{t}:i$ is visible to *a* }	The perception vector of agent *a* at time *t*
$H_{a,r}^{t}=\sum_{t\geq j\in T_{a,r}}g\left(a,s_{a}^{j},r\right)$	The foraging history of agent *a* and resource type *r* at time *t*
*T*_*a*,*r*_ = {*t* ∈ *T*: *a* choses to eat *r*}	The times at which agent *a* executes a foraging action on a resource of type *r*
$L\in N_{>0}$	The simulation length
$l\in N_{>0}$	The length of seasons
$H_{a}^{t}=\sum_{r\in R}H_{a,r}^{t}$	The foraging history of agent *a* at time *t*
$b:I\to R^{n}$	The behavior function which assigns a value to every action

### 2.1 Environment

The environment is modeled as a square grid of size *m* × *m* with continuous boundary conditions in which agents can move. Every grid cell can contain one of the two resource types, i.e. |*R*| = 2, whose proportions vary over time [28] such that in every “season” a specific resource is more abundant than the other.

#### 2.1.1 Food sources

The number of cells with resources, |*F*^*t*^|, is constant at every point in time: whenever one cell is emptied, a random quantity $\phi_{i,r}^{t}$ of resources of the same type spawns at a random location. New food sources are initialized to contain a random quantity of food, driven by the parameter Φ that determines the abundance of food.

#### 2.1.2 Seasons

The environment cycles periodically between two different configurations, named *seasons* [14, 28], which determine what resources are available for agents to forage. Foraging of different resource types is subject to trade-offs: the more an agent specializes in the gathering and consumption of one resource, the less effectively it forages the other resource, e.g. due to neophobia [29], a non-transferable skill set or other constraints, e.g. energy or memory constraints. This trade-off is modeled by a single *skill* parameter that determines the probability of success of foraging two resource types [30]. Environmental change is a known requirement for the evolution of learning, and seasons offer enough predictability for learning to be effective [31].

### 2.2 Agents

The agents serve as an abstract model for simple biological entities, which require to find food and forage in order to survive and reproduce. Agents are able to perceive their surroundings, i.e., defined as their range one Moore neighborhood, in the grid-like environment; the perception vector is denoted as I. A range one Moore neighborhood in a two-dimensional square grid is comprised of eight surrounding cells (horizontal (2), vertical (2) and two diagonals(4)).

Agent actions can either be a movement, that displaces them by one cell in the environment, or foraging, that consumes any available food in their current location. A foraging action fails if the current cell does not contain any resource, or randomly with probability $1-P_{f}\left(a,s_{a}^{t},r\right)$ otherwise (for agent *a* with skill level *s* at time *t* for resource type *r*).

#### 2.2.1 Aptitude and skill

The foraging strategy of an agent is determined by two parameters: (i) *aptitude*, which defines the value encoded in the genome and inherited from the parent, and (ii) *skill*, which defines the corresponding phenotypic expression and models the trade-off of specialization in a specific resource type [30] by influencing the probability of successful foraging.

For this reason, the skill of an agent is a determinant factor for the energy intake, their ability to reproduce, and consequently the fitness of the agents. The aptitude remains constant during the whole lifetime of an individual and changes only between generations via random mutations during reproduction. The initial value of skill at birth is determined by the inherited value of aptitude. If the skill parameter is plastic, i.e. adapts to the environment during the agent’s lifetime, then the value of aptitude influences only indirectly the energy intake of learning agents.

#### 2.2.2 Energy level

The energy level of an individual depends on three factors: (i) the availability of resources in the environment at each given time, (ii) the individual skill which determines the probability of successful foraging, and (iii) the individual behavior which determines what actions to execute for a given configuration of the environment.

More formally, the fitness function *f*(*a*, *t*) of an agent a∈A at time *t* is defined as the total energy intake:

$$f\left(a,t\right)=\sum_{t\in T}\epsilon H_{a}^{t}$$ (1)

Where $H_{a}^{t}$ is the foraging history and *ϵ* is the energy level increase factor.

Fitness depends on the foraging success function *g*:

$$g\left(a,s_{a}^{t},r\right)=\{\begin{array}{cc} 1 & \text{with}\;\text{probability}\;P_{f}\left(a,s_{a}^{t},r\right)\\ 0 & \text{otherwise}\; \end{array}$$ (2)

#### 2.2.3 Foraging, reproduction, death, and fitness

The experimental design introduces a trade-off between the foraging success of different resource types, determined by the skill $s_{a}^{t}$: agents can either become generalists, i.e. be able to forage both resources with a low probability, or specialize, i.e. be able to forage one resource with a high probability and lose the ability to forage the other.

Successful foraging increases the energy of an individual which determines the probability of reproduction. As agents compete for the same limited resources, efficient foraging translates to high reproduction rate.

The probabilities of foraging *P*_*f*_, reproduction *P*_*r*_ and of death *P*_*d*_ are defined as:

$$\begin{array}{c} \begin{array}{cc} P_{f}\left(a,s_{a}^{t}\right)= & {\left(s_{a}^{t}\right)}^{q}\\ P_{r}\left(a,t,c_{r}\right)= & f\left(a,t\right)/c_{r}\;\text{if}\;f\left(a,t\right)<c_{r}\;\text{else}\;1\\ P_{d}\left(a,t,c_{d}\right)= & d\left(a,t\right)/c_{d}\;\text{if}\;d\left(a,t\right)<c_{r}\;\text{else}\;1 \end{array} \end{array}$$ (3)

With a linear relation between skill and probability of foraging success, i.e. *q* = 1, the average total intake of an agent is equivalent to the average resource distribution: a specialist agent forages with certainty one type of resources but none of the other, while a generalist agent forages each resource with 50% probability. Assuming a non-linear relation between skill and foraging probability instead, i.e. *q* > 1, then a specialization leads to higher fitness than a generalization.

The effects of these values can be found in the supplementary material, S3–S5 Figs.

The framework determines the reproduction and death events by means of a genetic operator called *roulette wheel selection with stochastic acceptance* (as in Torney et al. 2011 [32]), according to which agents reproduce asexually with a probability *P*_*r*_ proportional to their fitness and die with a probability *P*_*d*_ proportional to their age. Upon reproduction, the energy level *ϵ* of the parent is split equally between the parent and the offspring and the offspring inherits a randomly-mutated copy of the parent’s genetic configuration.

### 2.3 Agent behavior

An agent’s desired behavior $B\left(a,t\right)={\text{arg}\;\text{max}}_{o\in O}\left(b\left(I_{a}^{t}\right)\right)$ associates the desired action to each perception vector I, containing a representation of the surroundings that informs about the location and presence of resources. This mapping between perception and action can be achieved by different techniques, e.g. an artificial neural network. The success of the desired action is determined by the *skill* value, which is defined as the phenotypic expression of the *aptitude* genotype.

The aptitude and the mapping *B*(*a*, *t*) changes from one generation to the next due to random mutations, and learning allows the inherited skill and the phenotypic expression of the mapping *B*(*a*, *t*) to be more suited to the current state of the environment.

#### 2.3.1 Agent types and learning

Two types of agents are introduced: reactive agents keep their behavior and skill constant throughout their lifetime, as they are a direct expression of the genotype, while learning agents adapt their behavior and skill according to their experience via reinforcement learning [16, 25, 26, 33–35]. Learning optimizes the expected reward associated with successfully foraging a resource of any type. Different reinforcement learning architectures are evaluated: Q-Learning [36], reinforcement learning based on a Restricted Boltzman Machine [37], Deep Reinforcement Learning [38] and reinforcement learning based on a single feed forward perceptron. The results presented in the main text are based on a single feed forward perceptron, see the supplementary material for further details, Section B in S1 File).

If learning is disabled (reactive agents), weights and skills cannot be learned and remain constant and equal to the inherited value for the whole lifetime of the individual, hence individuals are selected based on their inherited aptitude value. If learning is enabled, the behavior can adapt to changes in the environment. Specifically, the adaptation process happens through directly increasing the skill value after every successful foraging event and by using the successful foraging event as a reward signal in the reinforcement learning algorithm.

#### 2.3.2 Genotype and mutations

Upon reproduction, an offspring is generated that contains a mutated copy of the parent’s genome, consisting of the initial weights of the neural network, prior to any learning, and an additional gene called *aptitude*. These values are used to initialize the phenotype of the offspring.

#### 2.3.3 Reinforcement learning

Although, modeling biologically realistic entities is outside the scope of this paper; the study of the biological feasibility of different learning techniques including different versions of reinforcement learning, have shown that reinforcement learning is being able to reproduce certain human decision-making process and equilibrium [39–41]. More recently it has been shown that human level strategies can arise from reinforcement learning-based systems, even without human data [42]. In the case of this implementation of reinforcement learning, the behavior function of an agent *a* takes the form of $b\left(I_{a}^{t}\right)=Q\left(\cdot ,I_{a}^{t}\right)$ which indicates the Q-values for all actions and state $I_{a}^{t}$. The mapping between perceptions and actions is done via a neural network. Agents perceive their the environment, specifically, they are able to see a subset of the grid centered at their location (range one Moore neighborhood) and are able to identify food sources within this visual range, I. For the current model, a 3 × 3 region is observable and the food sources are observable but without the specificity of the amount of food contained. Based on this perception and using the neural network based choice model agents chose an action from their action space: move (north, south, east, west) or eat. Agents with different learning algorithms (Neural Network type) behave differently when faced with a variable environment, in terms of convergence and *adaptation to change* (see the supplementary material, Sections B and C in S1 File).

The agent skill is learned by increasing its value by Δ*S* after every time it performs foraging successfully, while for the choice of action the learning algorithms are based on the Reinforcement Learning approach, Q-Learning [36]. The Q-Table, a mapping from states/perceptions I and possible actions *O* to the quality value of each action for that state Q(I,O), of the original Q-Learning approach is replaced by a Q-Network as per [38] and the corresponding algorithm for the specific Q-Network structure is used for its training. The following equation describes the update to the quality values:

$$\Delta Q=(\overset{\text{learned}\;\text{value}}{\overset{︷}{\underset{\text{reward}}{\underset{︸}{r_{t-1}}}+\underset{\text{discount}\;\text{factor}}{\underset{︸}{\gamma }}\cdot \underset{O}{\text{max}}Q\left(I_{t},O\right)}}-\underset{\text{old}\;\text{value}}{\underset{︸}{Q(I_{t-1},O_{t-1})}})$$ (4)

$$\underset{\text{new}\;\text{desired}\;\text{value}}{\underset{︸}{Q(I_{t-1},O_{t-1})}}\leftarrow \underset{\text{old}\;\text{value}}{\underset{︸}{Q(I_{t-1},O_{t-1})}}+\underset{\text{learning}\;\text{rate}}{\underset{︸}{\alpha^{rlearn}}}\cdot \Delta Q$$ (5)

The results presented in the main text are based on Reinforcement learning using a single layer feed forward perceptron as its network architecture to “store” and query the Q-values, trained with backpropagation (PQL). The Q-network structure is b(I)=W·I+β where I is an input vector, *W* are the weights of the neural network and *β* the biases associated to the input layer. Further details about the variations depending on different neural network structures can be found in the supplementary material, Sections B and C in S1 File.

### 2.4 Measures

The degree of specialization of a population is measured with different metrics:

(I) the distribution of individual aptitudes across the population, according to which a higher frequency of extreme values corresponds to a more specialized population, (II) the individual foraging history, i.e. the frequency of successful foraging actions for a specific resource type, according to which extreme values indicate a specialized diet, (III) standard measures of group behavior that quantify the rate of consumption of resources ([43], page 241).

The degree of specialization of the population is measured by the distribution of aptitudes (I) at each given timestep, normalized by the population size at that timestep:

$$M^{I}\left(v,t\right)=|\left\{a\in A^{t}\;:\;s_{a}^{t}=v\right\}\left|/\right|A^{t}|$$ (6)

The foraging history (II) of the population at value *x* is measured as the frequency of individuals in the population who, during their lifetime, foraged a specific proportion of type *r* resources corresponding to *x*:

$$M^{II}\left(x,r\right)=|\left\{a\in A\;:\;H_{a,r}^{L}/H_{a}^{L}=x\right\}\left|/|A\right|$$ (7)

Additionally, standard measures of group behavior (III), taken from [43], page 241, are used to quantify the specialization of the population. The measures are defined and explained in the supplementary materials, Section H in S1 File.

While (I) measures the characteristics of the genotype, (II) and (III) measure the behavior of the agents which is determined by the phenotype.

## 3 Results: Computational model

### 3.1 The Baldwin effect

Previous work in the literature about the Baldwin effect found that the evolutionary process can be either speed up or slowed down [2] depending on the learning mechanism, the fitness function and the starting conditions of the population. Simulations are performed to verify whether or not the Baldwin effect exists in a cyclical environment, a question that, to the best of our knowledge, has not been answered before [17].

The existence of the Baldwin effect is evaluated by means of simulation by comparing the speed of genetic assimilation of phenotypic features as a function of the learning ability.

**Fig 1:**
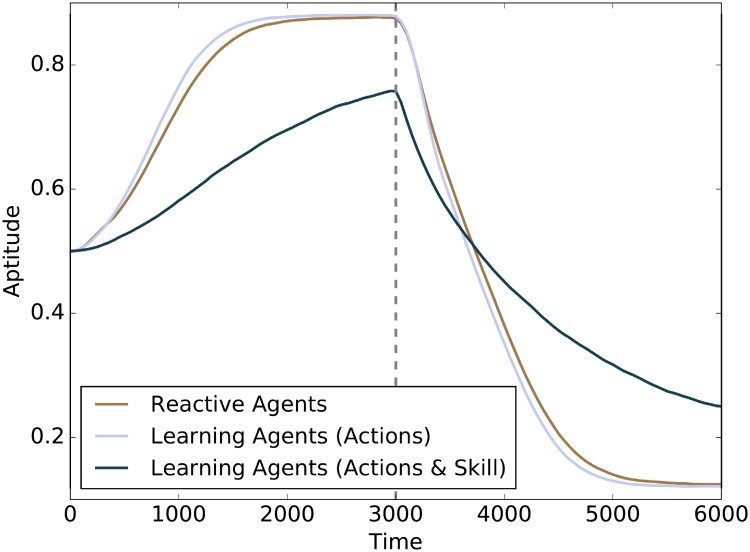
The Baldwin effect: Speed of genetic assimilation (genotype). Genetic assimilation of aptitude over time in two different learning populations, compared to a baseline of reactive agents. The simulations show that the speed of genetic assimilation changes with respect to the baseline, depending on the configuration of the learning algorithm, demonstrating that the Baldwin effect can speed up or slow down the genetic assimilation of aptitude. The dashed vertical line indicates a change of season, i.e. resource availability. Confidence intervals at the 95% confidence level are not shown as their size is negligible.

Fig 1 shows a comparison over time of three agent types in terms of the genetic assimilation of aptitude values due to changes in skill value:

- Reactive agents: baseline, i.e. unable to learn.
- Learning (Actions): agents that can modify their own actions through learning, a speedup in the genetic assimilation is observed.
- Learning (Actions & Skill): agents that can modify their own actions and their skill through learning, a slowdown in the genetic assimilation is observed.

The only difference between agent types concerns what traits can be learned. All other parameters of the learning algorithms are constant across types. The dependence of the speed of genetic assimilation on the degree of learning confirms the presence of the Baldwin effect.

### 3.2 A new effect: The Baldwin veering effect

This experiment investigates whether the Baldwin veering effect exists, i.e. a trait evolves by effect of plasticity that does not correspond to the plastic response induced by a cyclically-changing environment.

Slowly-changing environments allow populations to adapt via natural selection. Learning helps natural selection traversing the space of genetic configurations [44], and does so on a shorter timescale, therefore learning might speed up or delay this process. In quickly-changing environments, which change faster than the evolutionary timescale, learning and natural selection take on two different roles: Learning improves the behavior of agents in response to environmental variability, while natural selection improves the efficiency of learning.

The Baldwin veering effect is present if the following two conditions are verified: (i) the evolved trait differs from the environment-induced plastic response, i.e. genome and the phenotype converge towards different values, and (ii) this effect is determined by the presence of plasticity, i.e. the genetic configuration evolved by learning agents differ from that evolved under the same conditions by reactive agents.

Two genetic configurations are considered: a *specialist configuration* is defined as a genome whose aptitude evolves to one of the extreme values, i.e. specializes in either resource type, *generalist configuration* is defined as a genome whose aptitude evolves to an intermediate value. Different genetic configurations correspond to different initial learning efforts in terms of time required to adapt to the environment; assuming that an individual has the same probability of being born in either season, the optimal genetic configuration should reduce equally the effort of learning either skill.

**Fig 2:**
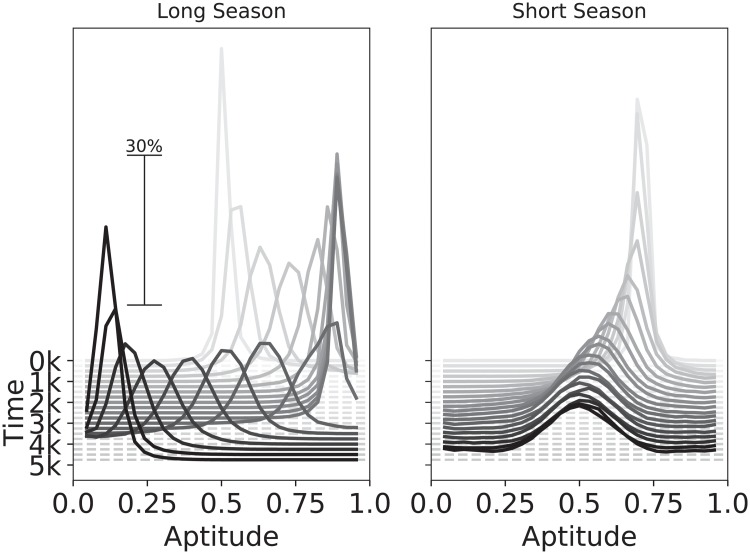
Comparison of aptitude distributions (genotype) of plastic agents in environments with different season length. The graphs show the change in the distribution of aptitude values across genomes of individuals in the population (horizontal-axis, Aptitude) over the course of the simulation (depth-axis, Time). Each point in the graph represents the frequency at which a specific aptitude value was present in the population (vertical-axis, % of the population) at a given time. The plot shows plastic agents during a long season (left) and short season (right) scenarios. During long seasons the population tracks the plastic induced response, while during short seasons the genetic trait evolves towards an intermediate value distribution. “k” denotes thousand abstract time-units.

The first condition is verified in Fig 2 by comparing the evolution over time of the inherited aptitude of populations of learning agents in a slowly-changing (Left) and in a quickly-changing environment (Right). Being able to quickly adapt to changes in the environment, the phenotype of learning agents tracks changes in resource availability, hence the induced plastic response is a specialized strategy corresponding to the most abundant resource type. In a slowly-changing environment, the genetic trait evolves towards the induced plastic response, while in a quickly-changing environment, the genetic trait evolves an intermediate value that corresponds to a generalist strategy which is not induced at the phenotypic level.

**Fig 3:**
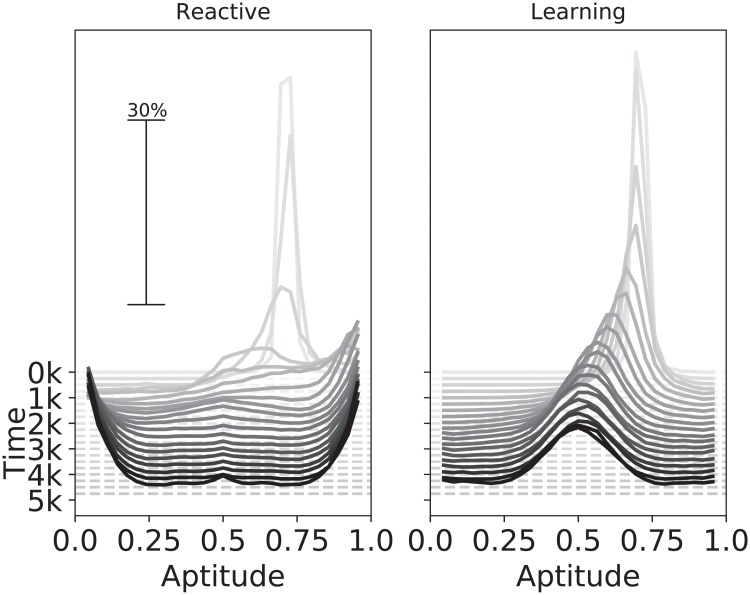
Comparison of aptitude distributions (genotype) between reactive and plastic agents in environments with short seasons. The graphs show the change in the distribution of aptitude values across genomes of individuals in the population (horizontal-axis, Aptitude) over the course of the simulation (depth-axis, Time). Each point in the graph represents the frequency at which a specific aptitude value was present in the population (vertical-axis, % of the population) at a given time. The left plot shows a population of reactive agents while the right plot a population of plastic agents, the populations evolve two different distributions, confirming that plasticity/learning can change the outcome of evolution. “k” denotes thousand abstract time-units.

**Fig 4:**
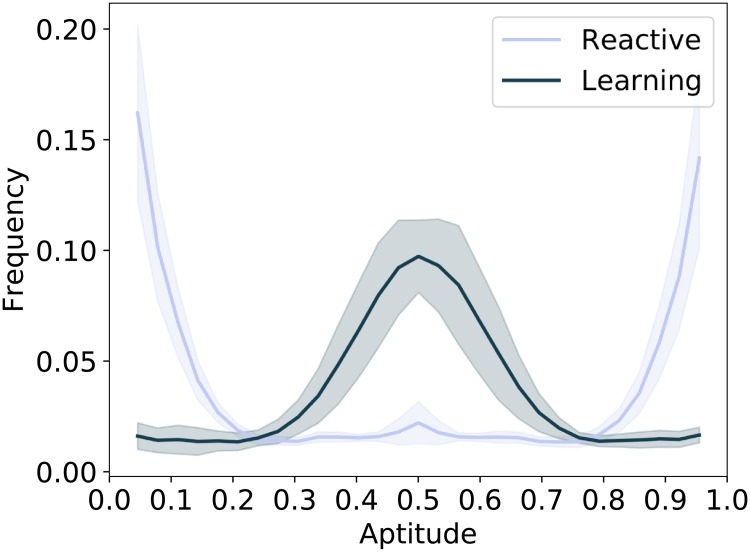
Comparison of final aptitude distribution (genotype) between reactive and plastic agents in environments with short seasons. The graphs show the average distribution of aptitude values (x-axis) across genomes of individuals in the population for the last 1000 iterations of the simulation, shaded areas represent confidence intervals at the 95% confidence level. The lines indicate the frequency at which a given aptitude occurs in the population. Plastic agents evolve a different distribution than reactive agents, confirming that learning can change the configuration to which evolution converges.

The second condition is verified in Fig 3 by comparing the evolution over time of the inherited aptitude of a population of reactive agents (Left) with that of a population of learning agents (Right). Both populations are initialized with an intermediate aptitude value, which evolves over time until it converges to some configuration after around 4000 timesteps. Reactive agents evolve extreme aptitude values, i.e. a specialist configuration. Specifically, half of the population evolves a high aptitude value (specialist in one type of resource) and the other half a low aptitude value (specialist in the other type of resource). The foraging success of reactive agents is determined directly by the static skill as inherited from the aptitude value, hence each half of the population specializes in foraging one or the other type of resource. For learning agents the foraging success is determined by the skill level, whose initial adaptation effectiveness is determined by the aptitude value. As a consequence, learning agents evolve an intermediate aptitude value, i.e. a generalist configuration, which allows them to adapt quickly to any environmental condition. Fig 4 highlights the difference between genetic configurations evolved by the two populations at the end of the simulation.

In the following section, we present further supporting evidence for these results.

### 3.3 Differences in individual behaviors

In order to verify that a difference in genetic configuration actually results in different behaviors, in this section, we analyze reactive agents instantiated with the genetic configurations of the agents that are alive during the last time-step of the previous simulations (see Fig 4). In order to produce a fair comparison, reproduction is also disabled, this way the only variable in the simulations is the genetic configuration which remains constant during these simulations and is expressed directly in the phenotype.

The goal of these new simulation set is to quantify the difference between genetic configurations evolved by different populations, this is achieved by evaluating the behavior that such configurations encode.

In these new simulations, the environment is set to have only one season and contains an equivalent quantity of both types of resources. An abundance of both resource types allows any foraging strategy to perform at its best, hence contributing to a fair comparison of different foraging strategies in terms of foraging success. The behavior of individuals is compared with the measures of foraging history and of group behavior, which are described in Section 2.4.

**Fig 5:**
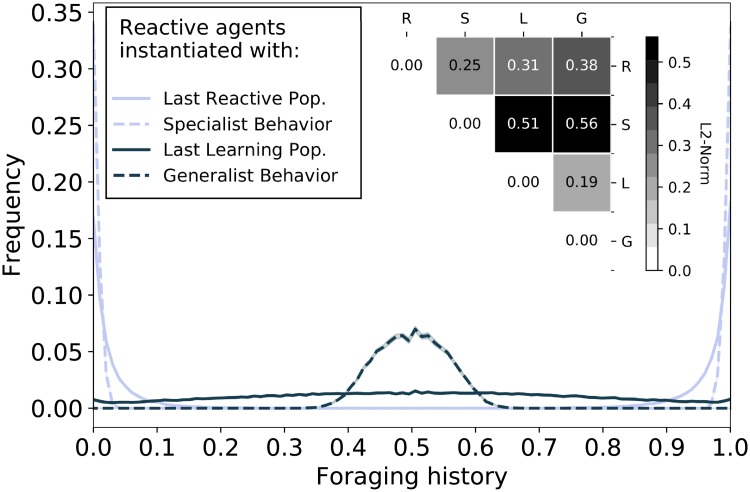
Comparison of foraging history (phenotype). The plots show the frequency at which a given value of foraging history occurs in the population. Foraging history is computed as the percentage of successful foraging actions or resources of type 0. A frequency of 0.2 associated to a value of foraging history of 0.4 means that 20% of individuals in the population foraged during their lifetime 40% of the time resources of type 0 and 60% of the time resources of type 1. In this simulation, all agents are reactive without the ability to reproduce and their genetic configuration is initialized from the distribution obtained from the second experiment (see Fig 4). Distributions of foraging actions resemble the distributions of aptitudes, confirming that different aptitude distributions produce different behaviors. Dashed lines represent baseline populations, where all agents have an aptitude value of 0.5 (generalist configuration) or half of the population has an aptitude value of 0.05 and the other half 0.95 (specialist configuration). Shaded areas (of negligible size) represent confidence intervals at the 95% confidence level. Inset is the *L*_2_-Norm between the different distributions.

Fig 5 shows the foraging history of the two populations of study. Additionally, it shows the foraging history of 2 baseline populations. These baseline populations are also reactive agents instantiated with genetic configurations specifically aimed to produce specialist behavior (being able to eat only one food type with high probability) and generalist behavior (being able to eat both food types with 50% probability). The foraging history shows that the behaviors in the two populations of study differ (cf. Fig 5), namely the population instantiated with the last reactive configuration is split into two groups of comparable size, each of which is specialized in foraging one type of resource, while the population instantiated with the last learning configuration has a more uniform foraging pattern which includes more generalists. The measure of individual foraging history is quantified by the frequency of foraging resources of type one, e.g. a value of 90% indicates that 90% of all resources foraged by the agent were of type one, and the remaining 10% of type two. These values are then aggregated across the population to determine the frequency of different values of foraging history. The inset of Fig 5 reports the *L*_2_ Norm between the distributions; learning configuration agents distribution is closer to the generalists’ distribution (0.19) while reactive configuration agents distribution is closer to the specialist distribution (0.25).

**Fig 6:**
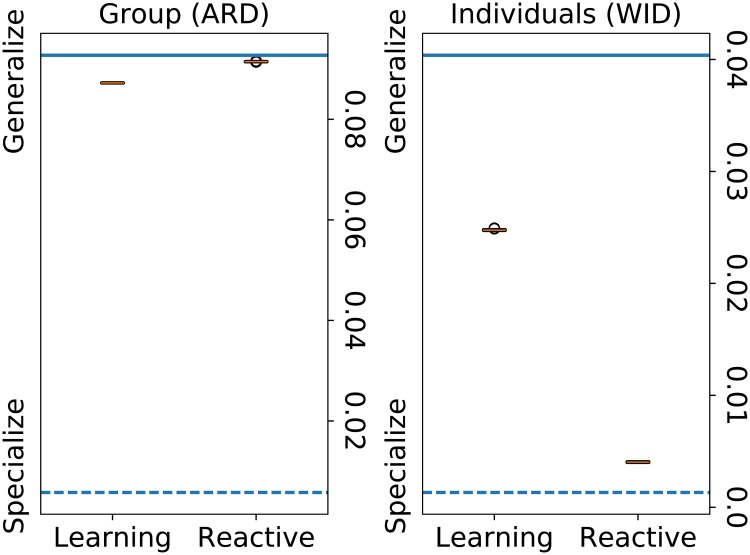
Measures quantifying the behavior of the population (phenotype). Left: among-resource diversity (ARD) quantifies the behavior of the population, both populations display a similar generalist behavior. Right: within-individual diversity (WID) quantifies the behavior of individual agents, learning agents behave more generalist than reactive agents. In this simulation, all agents are reactive without the ability to reproduce and their genetic configuration is initialized from the distribution obtained from the second experiment (see Fig 4). These results confirm that a difference in aptitude distribution corresponds to an actual difference in behavior. The solid line represents a baseline population in which all agents have an aptitude value of 0.5, the dashed line represents a baseline population in which each half of the agents have an aptitude value of 0.05 and 0.95 respectively.

Besides the measure of foraging history, different standard measures of group behavior [43] are used to compare the behavior of the populations (cf Fig 6). The interpretation of these measures is not straightforward, so baselines are added for reference: the dashed line represents the value of a population where half of the agents specialize in one resource and the other half in the other resource, while the continuous line represents a population of generalists.

The measures confirm that the learning configuration agents develop a generalist foraging strategy, both on the group level (among-resource diversity) and on the individual level (within-individual diversity). In contrast, last reactive configuration agents develop a more specialized foraging strategy on the group level (among-resource diversity). Understanding whether or not reactive configuration agents develop a specialized foraging strategy on the individual level is not straightforward, as a high value of among-resource diversity can either mean that different agents have different specialized diets or that agents have generalized diets. Combining this measure with that of within-individual diversity, which indicates a specialized diet on the individual level, allows us to conclude that specialization occurs also on the group level.

## 4 Results: Analytical model

The results outlined in the previous section showcase the existence of the Baldwin veering effect, but give little information about the process behind it. This section introduces and analyzes the predictions of an analytical model, inspired by previous work [45], which gives possible explanations to the simulation results and identify the conditions under which the Baldwin veering effect manifests. The model defines a fitness function for a generic individual, the evolutionary process is not explicitly modeled so evolutionary outcomes are inferred from considerations about the relative fitness of different individuals. Time and location of agents are not explicitly modeled, this abstraction is sensible because of the deterministic nature of seasonal changes, i.e. the environment displays the same conditions on average over each seasonal cycle. More fine-grained results about evolution and its dynamics might be obtained by pairing the fitness function with an existing model of evolution, e.g. [45, 46], such effort is outside the scope of this paper and is left for future work.

### 4.1 Description of the analytical model

The environment contains two types of resources, *j* = {0, 1}, whose proportion is denoted by *π*_0_ and *π*_1_.

The fitness *W*_*i*_ of a reactive agent *i* is formulated as follows:

$$W_{i}=\pi_{0}\cdot r_{i,0}+\pi_{1}\cdot r_{i,1}=\pi_{0}\cdot s_{i,0}^{q}+\pi_{1}\cdot s_{i,1}^{q}$$ (8)

Where the foraging success $r_{i,j}=s_{i,j}^{q}$ is determined by the agent’s skill *s*_*i*,*j*_ ∈ [0, 1] (which is equal to the aptitude level, being it a reactive agent) and by a parameter $q\in N_{\geq 0}$ which defines the relation between skill and foraging success. If the parameter *q* = 1, specializing on one resource and generalizing on two resources lead to the same foraging success. If *q* < 1 generalization becomes more beneficial than specialization as intermediate aptitudes produce a higher foraging success than extreme ones. Vice versa, specialization is more beneficial when *q* > 1 as the reward function is concave, a requirement for the co-existence of specialists and generalists in the same environment [47].

Following the design of the computational model, the two skills of an agent, as well as the resource proportions, are assumed to be complementary, i.e. *s*_*i*,0_ + *s*_*i*,1_ = 1, *π*_1_ + *π*_0_ = 1, therefore the notation can be simplified by defining *s*_*i*_ ≔ *s*_*i*,0_, 1 − *s*_*i*_ ≔ *s*_*i*,1_ and *π*_1_ = 1 − *π*_0_ which leads to:

$$W_{i}=\pi_{0}\cdot s_{i}^{q}+\left(1-\pi_{0}\right)\cdot {\left(1-s_{i}\right)}^{q}$$ (9)

**Fig 7:**
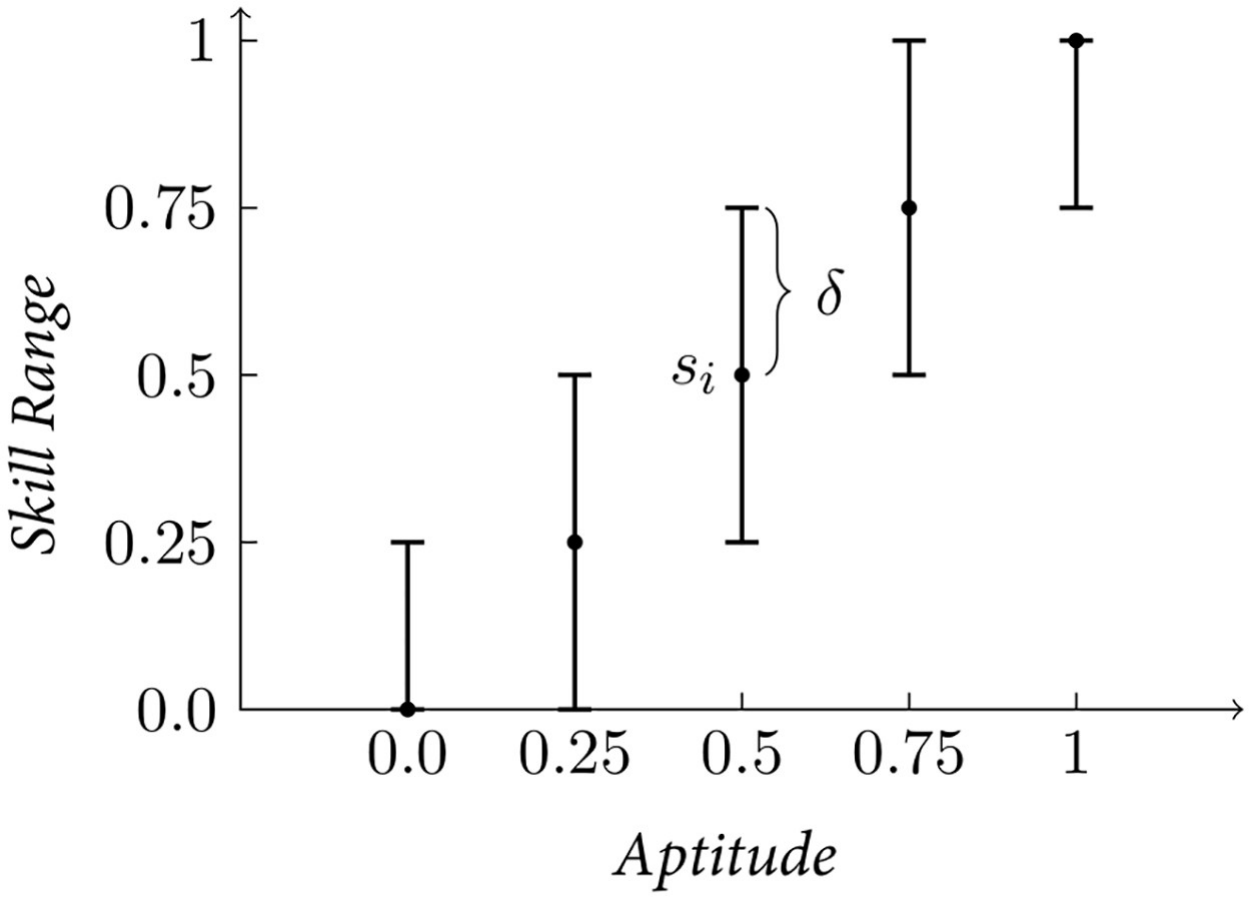
A sketch explaining the skill range *δ*. Skill ranges obtained with a fixed value of *δ* and different aptitudes.

In order to model learning agents, a new parameter *δ* is introduced which represents plasticity. The parameter *c* determines the cost of plasticity [48, 49]. A learning agent is not constrained by its inherited aptitude *α*, as its skill can adapt to changes in the environment. The value of *δ* determines the skills an agent can express by defining the maximum and minimum skill values: this range is centered on the aptitude and spans in both directions (cf. Fig 7), *s*_*i*_ = *α*_*i*_ ± *δ*. Given that the skill value is limited in the domain [0, 1], the previous expression for the bounds of skill values *s* is limited as follows, *s*_*i*_ = *min*(1, *α*_*i*_ + *δ*) for the beneficial side and *s*_*i*_ = *max*(0, *α*_*i*_ − *δ*) for the dis-favorable effect. For example an individual with aptitude 0.1 and *δ* = 0.6 can express any skill value in the range [0, 0.7]. As the aptitude is also constrained to the range [0, 1] the range of meaningful *δ* is also between [0, 1]. In this model, we consider only the effect of plasticity that increases the skill level (i.e learning that improves a skill):

$$W_{i}=\pi_{0}\cdot min{\left(1,\left(\alpha_{i}+\delta \right)\right)}^{q}+\left(1-\pi_{0}\right)\cdot min{\left(1,\left(1-\alpha_{i}+\delta \right)\right)}^{q}-c\cdot \delta$$ (10)

**Fig 8:**
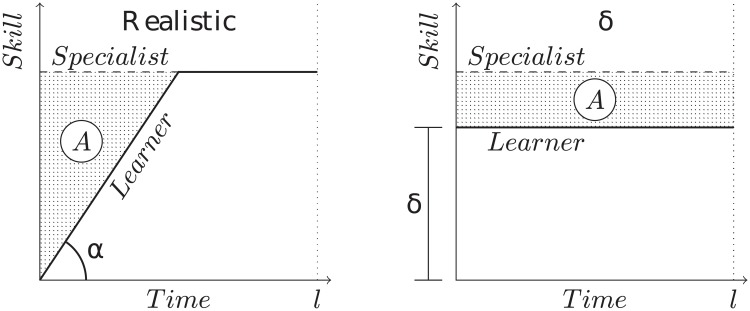
A sketch of modeling assumptions. The graph shows the change in skill level over time of a hypothetical learning individual. The shaded area represents the cost of adaptation: the loss in fitness caused by adapting to the environment with respect to an already adapted individual (specialist). Learning requires time to adapt, defined by the speed of learning *α*. This delay is modeled by reducing the plasticity *δ* such that the size of area *A* is the same.

For simplicity, the model assumes that agents adapt instantaneously to the environment by adopting the best available skill value *for each resource type*, i.e. skill of *s*_*i*_ = *α*_*i*_ + *δ* for resource type *π*_0_ and skill of *s*_*i*,1_ = *α*_*i*,1_ + *δ* = 1 − (*α*_*i*_ − *δ*) for resource type *π*_1_, which maximize the fitness function. The speed of learning, also called time lag, is modeled by reducing the value of *δ* (cf. Fig 8). In practice the value of *δ* depends on the ratio between the speed of learning and the season length: a slower learning mechanism reduces the distance to which the value can change, similarly, a shorter season reduces the number of experiences an agent has during a season.

### 4.2 Analysis: Baldwin veering effect

**Fig 9:**
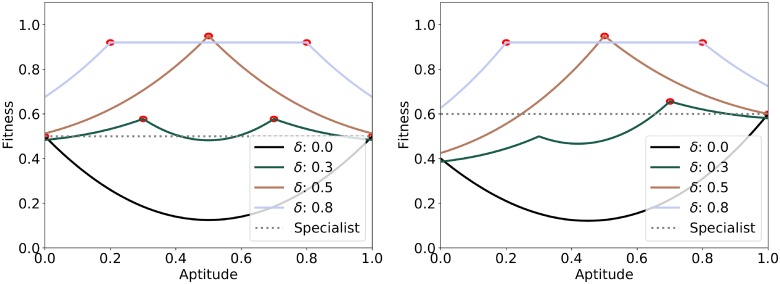
Fitness corresponding to different combinations of aptitude and *δ*, for *q* > 1 and *c* > 0. The plot shows the fitness predicted by the analytical model for given values of aptitude and *δ*. Left: *α*_0_ = 0.5, right: *α*_0_ = 0.6. The red circles represent the maximum fitness achievable for a given value of *δ*, i.e. the attractors of the evolutionary process. Increasing the value of *δ* from 0 to 1, the optimal aptitude values start at the extremes (0,1) and move towards the center as *δ* increases. The maximum fitness is obtained for *δ* = 0.5, where there is only one maximum for an aptitude of 0.5. For values of *δ* > 0.5 a range of aptitudes, centered on and expanding from 0.5, maximizes the fitness. The dotted line corresponds to the fitness of a specialist individual, which becomes lower than the fitness of learning individuals as values of *δ* increase. Also note that the introduction of learning, i.e. *δ* > 0, changes the aptitude for which fitness is maximized, i.e. the configuration towards which evolution converges.

Fig 9 shows how different aptitudes compare, in terms of fitness, for varying values of plasticity *δ*. A combination of aptitude and plasticity associated with a higher fitness value produces more fit individuals that are favored by natural selection. The red circles represent the globally optimum aptitudes for a given value of *δ*, i.e. the attractors in genetic configuration space of the evolutionary process. If *δ* < 0.5 agents evolve a specialist configuration, as opposed to a generalist configuration if *δ* = 0.5. Note that the configuration with *δ* = 0.5 and aptitude *α*_*i*_ = 0.5 maximizes the fitness as it allows agents to choose any skill value in the range [0, 1], hence allows agents to forage both resource types with certainty. This condition is observable in the agent-based model simulation when the speed of learning is as fast as the frequency of change in the environment, i.e. an agent adapts its skill to a new environmental state but does so too slowly to remain specialized for a long time before the environment changes again. This confirms the existence of the “Baldwin veering effect”, as any value of *δ* > 0 changes the fitness landscape such that fitness is maximized by a different aptitude, which is then selected. These results hold even for asymmetric seasons, i.e. when the probability of one season is higher (cf. Fig 9 right).

For values of *δ* > 0.5, learning makes an increasingly large range of aptitude values equivalent in terms of evolutionary fitness which could allow agents to generalize, but such a configuration would not evolve in reality as the overall fitness is reduced when compared to *δ* = 0.5. These results are confirmed also for *c* = 0 and *q* ≤ 1, see the supplementary material, Section G in S1 File.

Concluding, learning agents evolve an intermediate aptitude, i.e. a generalist configuration, only if learning speed is proportionate to the season length such that agents can adapt to both resource types. This result is general and holds independently of the value of *q* and resource proportion *π*_0_, hence confirms that the Baldwin veering effect depends exclusively on the timescales of learning and environmental change.

## 5 Discussion

A common finding in the literature about the interactions between plasticity and cyclically changing environments is that plastic individuals, who can adapt to changes in the environment after a certain time lag, i.e., speed of learning, are more fit than non-plastic individuals, who are unable to adapt, when the frequency of change in the environment is faster than a certain threshold. The definition of plasticity varies in the literature: plasticity is modeled as switching between two distinct phenotypes [14, 50], as a change in niche breadth [51], or—as in this work—as behavioral adaptation through learning [52, 53]. Related work concludes that similar patterns of specialization and generalization in the phenotype might develop also when assuming non-reversible plasticity, i.e. a phenotypical trait can assume only one specialized state in an individual’s lifetime [19]. Although this work focuses on reversible plasticity, i.e. the same phenotypical trait can change from one specialized state to another, reversibility is not claimed to be a prerequisite for the existence of the Baldwin veering effect. Our claim about the existence of the Baldwin veering effect is not invalidated by whether or not the effect manifests also in the presence of non-reversible plastic traits, this is nevertheless a promising research question for future work.

It is important to note that although the concepts of specialization, i.e. adaptation to only one state, and generalization, i.e. trading-off adaptation across more than one state, are consistent across the literature, the concepts of generalist and specialist can differ substantially: while the definition of specialists adopted by this work implies the ability to specialize in only one resource, unless the agent is able to learn, other work defines them as able to specialize simultaneously on many resources [14]. To the best of our knowledge, this work is the first to investigate the effect of plasticity and cyclical changes in the environment on the evolution of the genetic configuration. Although, [54] considers local variation (cycles) the analysis is focused on a single movement to an extreme environment; our work is consistent in terms of the expected genetic assimilation. Previous work investigates the evolution of plasticity by analyzing the co-evolution of populations with different genetic configurations [55, 56], or by analyzing the scaling of plasticity itself [54] while in the current work traits are either plastic or not. Due to the differences between our models, we cannot make more extensive claims, i.e. regarding the development of co-evolving populations nor the evolution of plasticity itself. However, results pertaining to the more general aspects of the Balding effect, i.e. genetic assimilation, is consistent across the works. Other work [14] predicts that non-plastic individuals would evolve a genotype that leads to a wide tolerance function, making the individual able to adapt to a broader set of environmental configurations. This is in conflict with our finding that non-plastic individuals specialize in one environmental configuration, which leads to a split in the population. We believe this difference is caused by a modeling assumption that is relaxed in this paper, i.e. each agent can express a different phenotype (tolerance function) for each environmental state. The thread of literature looking at the evolution of artificial neural networks [52, 57] concludes that different levels of plasticity lead to the evolution of different weights. The main difference with the proposed model is that the environment changes [52] during an individual’s lifetime [57], hence that model is not able to capture the effect of the frequency of environmental change on evolution which is crucial for the results presented in this work.

The results presented in this work rely on the assumption that information about the environment is always precise. Relaxing this assumption requires the consideration of imperfect perceptions. Hence, the agents need to learn an estimate of the environmental state, before they can begin phenotypic adaptation [58] or while they are adapting [19]. Previous work finds that agents with imperfect perception learn accurate estimates of the probability distribution of environmental states and demonstrates genetic assimilation of phenotypic features, i.e. the Baldwin effect [59]. These results suggest that the Baldwin veering effect does not depend on the assumption of perception accuracy.

The aim of this paper is to provide a proof of concept, not modeling realistic entities, hence the model is constrained to only two resources. Increasing the complexity of the environment, as well as introducing group behavior, is required to model any realistic ecosystem and is left for future work.

The Baldwin veering effect can be interpreted as the interaction between two different traits throughout the evolutionary process. This interpretation could be described as a co-evolutionary process between two different traits in the same genotype: (1) the evolutionary selection pressure on the existence of plasticity implies a specific evolutionary selection pressure on (2) the aptitude level.

This effect can also be interpreted as an extended form of gene interactions [18] that affects both the phenotype and the genotype.

Plastic behavior is the outcome of complex interactions between genes, for the sake of tractability, this work abstracts these interactions as the effect of one single gene called aptitude. This simplification is reasonable to model some simple natural organisms e.g. fish behavior [60] and foraging in bacteria [61]. Gene interaction has an effect on the model, i.e. the interaction between the aptitude and the gene for plasticity changes the phenotypic expression of individuals [18]. Nevertheless, the results are more than just a special case of gene interaction as the presence of a “plasticity gene” causes changes both at the phenotypic and at the genotypic level, i.e. a different genetic code evolves in the population, which in turn produces different phenotypic traits.

Although this suggests the existence of the hypothesized effect, the theory does not clarify what processes cause learning agents to evolve a generalist configuration instead of a specialist configuration. One possible mechanism is that a generalist configuration allows individuals to have a more constant foraging success than a specialist configuration, as a skill level that oscillates around an average value allows individuals to forage more or less constantly throughout their lifetime, while a skill level oscillating around any of the extremes would result in periods of high and periods of low foraging success. An imbalance in foraging success translates to higher variance in offspring number, which is known to reduce the fitness [62]. Another possible mechanism for the evolution of a generalist configuration would be to provide indirect rewards: then, an intermediate aptitude would increase the evolutionary fitness indirectly by allowing for a faster adaptation to any environmental configuration. A similar mechanism has been described in the literature about intrinsic motivation, where evolution favors actions that are providing rewards only indirectly. For example, it has been found that curiosity and playfulness at a young age can improve fitness at a later age [63]. Understanding what processes cause the evolution of a generalist configuration is a worthy result in its own right which should be addressed in future work.

Another relevant observation is one of the convergent phenotypic outcomes. This phenomenon is highlighted in the fact that the neural network weights defining the desired behavior are initialized randomly and individuals with different genetic composition (weights) converge to similar behaviors but different weight composition. This shows that the large space of weights combinations posses a large number of equivalent optimums. Further studies in this topic might provide deeper insights relevant to the machine learning community.

Future work will also verify the predictions of the analytical model within the agent-based simulation framework, in particular, that there exists a configuration for which a learning population splits into two groups of specialists with aptitude values in [0, 0.5] and [0.5, 1] respectively, and a configuration in which learning population evolve a uniform distribution of aptitude values.

## 6 Conclusions

Plasticity, e.g. learning, is known to influence the speed at which evolution converges to some “target” configuration. This work, in contrast, addresses the question of whether or not plasticity in a cyclically changing environment can lead to the evolution of a different genetic configuration. Following previous work, this question is answered by means of an agent-based model of a foraging task, with cyclical variability in the resource distribution. Additionally, this result is confirmed through an analytical model.

Experimental and analytical results show the existence of the Baldwin effect in a cyclical environment and identify the novel “Baldwin veering effect”, i.e. a trait (generalist configuration) evolves by effect of plasticity that does not correspond to the plastic response induced by a cyclically-changing environment (specialist configuration) and the conditions under which it exists. A mathematical model verifies that the introduction of plasticity in the phenotype changes the fitness landscape in a way such that a generalist configuration becomes the global optimum in the space of genotypes.

These results are relevant for the literature of Evolutionary Biology, as they expand the understanding of how phenotypic plasticity influences evolution and present a novel effect caused by the interaction between learning and evolution. These results might also help to understand the effect of a fast learning process on a slow learning process in another context, which has a cyclical component, for example, opinion formation in settings where learning [64, 65] mediates the rate of exposure to different opinions [66, 67].

### 6.1 Data availability

The source code used to generate and analyze the data-sets is available on GitHub [68, 69]. Other information, including the parameters and libraries used, is provided in the Supplementary information, see Sections D and E in S1 File.

## Supporting information

S1 Fig Comparison of different learning algorithms. Each graph represents the frequency over time of an agent choosing to forage each resource type whenever the corresponding resource is available. A higher value produces a higher fitness, assuming the corresponding resource is available in the environment. Each curve is the average of 300 independent simulations. Season length is 3000 and all simulations start in the same season.
(EPS)

S2 Fig The genetic configuration evolved with different learning algorithms. Top left: PQL. Top right: QL. Bottom Left: RQL. Bottom right: DRL. All algorithms are able to reproduce the main result of the paper, i.e. the evolution of a generalist configuration.
(EPS)

S3 Fig Fitness for different combinations of aptitude level and *δ* for *q* > 1 and plasticity cost *c* = 0. Note that values of *δ* > 0.5 now maximize the fitness so an evolutionary outcome is possible where a mix of specialists and generalists co-exist. Left: *a*_0_ = 0.5, right: *a*_0_ = 0.6.
(EPS)

S4 Fig Fitness for different combinations of aptitude level and *δ* for *q* = 1 and plasticity cost *c* = 0. Intermediate aptitude levels deliver the same fitness as extreme levels, thus a mixed population will evolve. An intermediate aptitude level of 0.5 is optimal if *δ* = 0.5, while an extreme aptitude level is optimal for high or low values of *δ*. Left: *a*_0_ = 0.5, right: *a*_0_ = 0.6.
(EPS)

S5 Fig Fitness for different combinations of aptitude level and *δ* for 0 < *q* < 1 and plasticity cost *c* = 0. Intermediate aptitude levels deliver higher fitness than extreme levels, hence specialists have always a lower fitness than generalists. An intermediate a aptitude level is optimal in any circumstances. Left: *a*_0_ = 0.5, right: *a*_0_ = 0.6.
(EPS)

S1 File Supporting information. Document containing supplementary material.
(PDF)
