# Supplementary material for: How learning can change the course of evolution
Source: PLoS One. 2019 Sep 5;14(9):e0219502. doi: 10.1371/journal.pone.0219502 (PMC6728028; doi:10.1371/journal.pone.0219502)
Supplement: S1 File — Document containing supplementary material. (PDF) [file pone.0219502.s006.pdf]

How learning can change the course of evolution. Supplementary information

Leonel Aguilar[1,¶,*], Stefano Bennati[1,¶], Dirk Helbing[1,#a]

1 Professorship of Computational Social Science, ETH Zürich, Zürich, 8092, Switzerland

[#a]Current Address: Professorship of Computational Social Science, ETH Zürich, Zürich, Switzerland

[*]leonel.aguilar@gess.ethz.ch (L.A.)

[¶]S.B. and L.A. are Joint First Authors

A Computational model

This section describes computational experiments highlighting different effects of learning in evolution.

Computational experiments are performed on a population of agents foraging in a dynamic environment under the effect of natural selection. The environment is made dynamic with the introduction of seasons that differ in the proportion of resources present in the environment, e.g. only one type of resource is produced in every given season.

B Learning

This section discusses how different learning algorithms behave when faced with a variable environment, in terms of convergence and *adaptation to change*. The skill gets increased by ΔS after every successful foraging event, while for the action the learning algorithms are based on the Reinforcement Learning approach, Q-Learning [1]. The

Q-Table, a mapping from states/perceptions $\mathcal{I}$ and possible actions O to the quality value of each action for that state $Q(\mathcal{I}, O)$, of the original Q-Learning approach is replaced by a Q-Network as per [2]; using the following equation (B Equation) and a corresponding training algorithm for each Q-Network structure.

$$\Delta Q = \left(\overbrace{\underbrace{r_{t-1}}_{\text{reward}} + \underbrace{\gamma}_{\text{discount factor}} \cdot \max_O Q(\mathcal{I}_t, O)}^{\text{learned value}} - \underbrace{Q(\mathcal{I}_{t-1}, O_{t-1})}_{\text{old value}} \right) \tag{A}$$

$$\underbrace{Q(\mathcal{I}_{t-1}, O_{t-1})}_{\text{new desired value}} \leftarrow \underbrace{Q(\mathcal{I}_{t-1}, O_{t-1})}_{\text{old value}} + \underbrace{\alpha^{rlearn}}_{\text{learning rate}} \cdot \Delta Q \tag{B}$$

We name the different reinforcement learning algorithms based on their Q-Network structure:

- PQL: Reinforcement learning using a single layer feed forward perceptron as its network architecture to "store" and query the Q-values, trained with backpropagation.
- RQL: Reinforcement learning using a variation of a Restricted Boltzmann machine [3] for the network architecture, trained with contrastive divergence.
- Q-Learning [1], trained by directly replacing the Q-values in the Q-table. DRL: Deep Reinforcement Learning [2]: using 3 fully connected layers:

  1. $(perception\_size \times perception\_size * 5)$
  2. $(perception\_size * 5 \times number\_of\_actions * 5)$
  3. $(perception\_size * 5 \times number\_of\_actions)$

  The DRL implementation uses experience replay with a memory replay of 50 experiences and is trained using back-propagation. The use of experience replay improves DRL's learning convergence.

The Q-network structure in presence of an input vector $\mathcal{I}$ takes the form of:

1. PQL: $b(\mathcal{I}) = W \cdot \mathcal{I} + \beta$ where W are the weights of the neural network and β the biases associated to the input layer.
2. RQL: $b(\mathcal{I}) = \sigma(W \cdot \mathcal{I} + \beta)$ where σ denotes the logistic sigmoid.

3. Q-Learning: $b(\mathcal{I}) = Q(\mathcal{I})$ where Q is the Q-table, i.e. a value table.

4. DRL: $b(\mathcal{I}) = G^3 \circ G^2 \circ G^1$ where $G^L(x) = \sigma(W^L \cdot x + \beta^L)$.

Agents perceive their the environment, i.e. they are able to see a subset of the grid centered at their location and are able to identify food sources within this visual range, $\mathcal{I}$. For the current model, a 3×3 region is observable and the food sources are observable but without the specificity of the amount of food contained. Based on this perception agents are able to perform an action either: move (north, south, east, west) or eat.

The results of each learning algorithm are the average of 300 independent simulations, parameters are consistent across simulations.

Results show that different types of learning algorithms have different speeds of convergence (cf. S1 Fig) shows the proportion of agents choosing to eat while a specific type of resource is in their foraging range. Some learning algorithms adapt faster than others to changes in the environment.

RQL is the fastest to adapt to a change in the environment, and it also shows a stronger tendency to forget the learned behavior in the opposite season. DRL is the slowest to learn. This is not surprising as deep networks are generally trained with large datasets and used for much more complex tasks.

C The Baldwin Veering Effect and the learning algorithm

In order to analyze the consistency of the results in respect to the type of learning, learning algorithms are compared by reproducing the main result of the paper, i.e. the evolution of a generalist configuration (cf. S2 Fig). Different learning algorithms produce different features in the genetic configuration, for example, QL has a lower variability than PQL, and both RQL and DRL appear to have a trimodal distribution where some specialized individuals co-exist with generalist individuals. Nevertheless, the genetic configuration produced by all learning algorithms features a clear peak for aptitude of 0.5, indicating the presence of generalist individuals, hence supporting the main result of the paper, i.e. the existence of the Baldwin veering effect.

Parameter	Symbol	Value	Description
Initialization			
num-agents	N^0	100	The size of the initial population.
skill-level	s_a^0	0.7	The average aptitude level of the initial population.

Table A. Description of the parameters in the model and their value. Initialization

Parameter	Symbol	Value	Description
Environment			
field-size	m	20	The size of the grid.
max-food	Φ	50	The maximum resource quantity that a cell can contain.
num-food	$\|F^0\|$	400	The number of cells containing some food.
food-proportion	F_0^0/F_1^0	1.0	The proportion of the 'seasonal' resource with respect to the total amount of resources.
food-energy	ϵ	10	The energy given by a unit of resource.

Table B. Description of the parameters in the model and their value. Environment

PQL has been chosen as the learning algorithm for the experiments presented in the paper, as it offers a good compromise between capacity and computational requirements.

D Parameters of the computational model

The following tables show the values of the parameters used for the computational experiments.

E Reproducibility

A C++ compiler with OpenMP support is required in order to compile the code. OpenMPI is used for the parallel computing extension. Other requirement is tiny-dnn [4], used for the reinforcement learning algorithms. The code has been compiled with Make and the GCC compiler (see F Table). Other development environments and libraries might be compatible as well. Data analysis and figures are

Parameter	Symbol	Value	Description
Agent			
max-age	c_d	1000	Age after which the probability of death is 1. (figure 1. used 3000)
max-energy	c_r	max-age	Age energy after which the probability of reproduction is 1.
fov-radius	$\sqrt{I}//2$	3	The range of the Moore neighborhood where the agent can perceive.

Table C. Description of the parameters in the model and their value. Agent parameters

Parameter	Symbol	Value	Description
Learning			
algorithm	B	PQL	Reinfocement learning using a single layer perceptron as the Q-table and Back propagation to train the network (learning)
alpha	α^{rlearn}	1	Learning rate
gamma	γ	0.5	Discount rate
epsilon	ϵ	0.1	Percentage of exploratory actions
reward-energy	r_t	1	Positive reinforcement for successful foraging.

Table D. Description of the parameters in the model and their value. Learning

Parameter	Symbol	Value	Description
Simulation			
sim-length-f1	L	6001	The simulation length in fig. 1 main text
season-length-long	l	3000	The length of a long season
sim-length-other	L	5001	Length of the simulation
season-length-short	l	50	The length of a short season
max-agents	N	2000	The maximum population size, enforced by killing random agents in surplus.
samples		300	The number of independent simulations.

Table E. Description of the parameters in the model and their value. Simulation

produced with Python (Pandas, Matplotlib). Compilation and startup scripts are written for bash on a *nix system, but other shells might be supported as well. The code has support for the LSF platform for parallel execution on clusters, but it can also be run on a single machine. Simulations complete in a reasonable time: A simulation with 20,000 agents runs on a cluster node with 24 CPU-cores takes less than 24 hours with shallow reinforcement learning algorithms (PQL, RQL, QL) and less than 120 hours with deep reinforcement learning algorithms.

Flag	Description
debug	activates debug prints
invisible_food	food cannot be seen at a distance
immortals	disables evolutionary process (birth and death)
nonlinear_prob	Proportion between skill and foraging probability is non-linear
Learning	
learn	enables learning
brain_ql	selects QL as learning algorithm
brain_pql	selects PQL as learning algorithm
brain_rql	selects RQL as learning algorithm
brain_deep	selects DRL as learning algorithm

Table F. Description of compile flags.

F Analytical model assumptions.

The analytical model relies on restrictive macroscopic assumptions which enable a straight forward analysis:

- The fitness of agents is modeled over an abstraction of individual cycles (periods of two seasons that repeat) that removes the time component.
  - Available resources are assumed to be constant and equal to the average over a cycle.
  - Agents do not move, instead, they access resources of types 0 and 1 with probabilities π_0 and π_1 respectively.
  - *Evolution* is not modeled explicitly, instead, the evolutionary outcome is inferred from the fitness levels obtained within each cycle.
- Learning is modeled as skill level plasticity (aptitude + δ): the parameter δ determines the range of skill levels an agent can choose at the start of the cycle.

G Analytical model: edge cases

In this section, we provide further observations regarding the analytical model. Equation C reproduces equation 3 from the main text.

$$W_i = \pi_0 \cdot min(1, (\alpha_i + \delta))^q + (1 - \pi_0) \cdot min(1, (1 - \alpha_i + \delta))^q - c \cdot \delta \tag{C}$$

Where the parameters α_i, δ, π, q can assume values in the interval $[0, 1]$.

Considering the case where $c = 0$, i.e. plasticity has no cost, any increase in δ provides an increase in fitness, bounded by the cases where $a_i + \delta \geq 1$ and $1 - a_i + \delta \geq 1$. If $\pi_0 = 0.5$ the maximum fitness is reached when both bounds are reached simultaneously,

$$(\alpha_i + \delta) = 1 - \alpha_i + \delta \tag{D}$$

$$2 \cdot \alpha_i = 1 \tag{E}$$

$$\alpha_i = 0.5 \implies \delta = 0.5 \tag{F}$$

The maximum fitness of 1 is reached for $\alpha_i = \delta = 0.5$ and keeps the same value, 1, for any values of $\delta \geq 0.5$, and $\alpha_i + \delta \geq 1$. If $\pi_0 = 1$ or $\pi_0 = 0$ the maximum bound is reached for any values of α_i and δ such that the bounds $\alpha_i + \delta \geq 1$ or $1 - \alpha_i + \delta \geq 1$ are satisfied respectively.

In the case where $c > 0$, i.e. plasticity has a cost: Given a combination of α_i, δ and π that reached the maximum value in equation 3, any further increase in δ would result in a decrease in fitness.

G.1 Analytical model sensitivity to different values of q when $c = 0$

The results presented in the main text are validated here in absence of plasticity costs, i.e. $c = 0$, and for different values of q.

From S3 through S5 Fig we can observe that qualitatively similar results are produced also for $q = 1$ and $0 < q < 1$.

H Diversity measures for social foraging

Assume a group contains G individuals and S discrete resource types.

- n_{gs} is the number of items of resource s consumed by individual g.
- $n_{g.} = \sum_{s=1}^{S} n_{gs}$ is the total foraging of individual g.
- $n_{.s} = \sum_{g=1}^{G} n_{gs}$ is the number of resources of type s foraged by any agent.
- $n_{..} = \sum_{g=1}^{G} \sum_{s=1}^{S} n_{gs}$ is the number of resources of any type consumed by any agent.

Each $n_{gs} > 0$ defines a sample proportion p_{gs} where $p_{gs} = n_{gs}/n_{..}$, which is used to estimate the total, cross-classified diversity:

$$h'(g \times s) = -\sum_{g=1}^{G} \sum_{s=1}^{S} p_{gs} ln(p_{gs}) \qquad \text{(G)}$$

The following measures of social foraging [5, Pag. 241] are based on the concept of diversity [6]:

A generalized diet includes most of all resources types in roughly equal proportions. A specialized diet includes one or a few resource types at high proportions, and very low proportional levels of the remaining resources. The group's diet refers to the pooled resource consumption of all group members.

- Among-resource diversity $h'(s) = -\sum_{s=1}^{S} p_{.s} ln(p_{.s})$
  - Low: group specializes because individuals have similar specialized diets
  - High: group generalizes, individuals may generalize or different individuals have different specialized diets.
- Average within resource diversity $E[h'(g|s)]$.
  - Low: different individuals have different specialized diets, so group generalizes; a similar effect occurs whenever different individuals consume different total amounts of resources.
  - High: individuals have similar diets, whether generalized or similarly specialized, group diet may then be generalized or specialized.
- Among-individual diversity $h'(g)$.
  - Low: individuals differ in amount of resources consumed, independently of each individual's specialization or generalization.
  - High: Individuals consume similar amounts of resources, independently of each individual's specialization or generalization.
- Average within-individual diversity $E[h'(s|g)]$.
  - Low: Individuals specialize independently, group may consequently specialize or generalize.
  - High: individuals generalize, group consequently generalizes.

Table G. Reproduced from [5, Pag. 241]

- Among-resource diversity: $h'(s) = -\sum_{s=1}^{S} p_{.s} ln(p_{.s})$
- Conditional phenotypic diversity within resource s:

$$h'(g|s) = -\sum_{g=1}^{G} (\frac{p_{gs}}{p_{.s}}) ln(\frac{p_{gs}}{p_{.s}})$$

- Average within-resource diversity: $E[h'(g|s)] = \sum_{s=1}^{S} p_{.s} h'(g|s)$
- Among-individual diversity: $h'(g) = -\sum_{g=1}^{G} p_{g.} ln(p_{g.})$
- conditional resource-consumption diversity:

$$h'(s|g) = -\sum_{s=1}^{S} (\frac{p_{gs}}{p_{g.}}) ln(\frac{p_{gs}}{p_{g.}})$$

- $E[h'(s|g)] = \sum_{g=1}^{G} p_{g.} h'(s|g)$

References

1. Watkins CJ, Dayan P. Q-learning. Machine learning. 1992;8(3-4):279–292.
2. Mnih V, Kavukcuoglu K, Silver D, Rusu AA, Veness J, Bellemare MG, et al. Human-level control through deep reinforcement learning. Nature. 2015;518(7540):529–533.
3. Hinton G, Osindero S, Welling M, Teh YW. Unsupervised discovery of nonlinear structure using contrastive backpropagation. Cognitive science. 2006;30(4):725–731.
4. tiny dnn. Header only, dependency-free deep learning framework in C++14; 2018.
5. Giraldeau LA, Caraco T. Social foraging theory. Princeton University Press; 2000.
6. Patil G, Taillie C. Diversity as a concept and its measurement. Journal of the American statistical Association. 1982;77(379):548–561.
